# Diffuse Neurofibroma in a Patient with Unknown History of NF1

**DOI:** 10.1155/2018/8768472

**Published:** 2018-06-10

**Authors:** Mahmoud A. K. Ebrahim, Mishal M. AlMutairi, Khaled M. Hindi, Jassem M. Bastaki

**Affiliations:** ^1^Farwaniya Hospital, Ministry of Health, Kuwait City, Kuwait; ^2^Zain Hospital, Ministry of Health, Kuwait City, Kuwait; ^3^Kuwait Institute Medical Specialties, Kuwait City, Kuwait; ^4^Al-Sabah Hospital, Ministry of Health, Kuwait City, Kuwait

## Abstract

Malignant peripheral nerve sheath tumor (MPNST) is a rare disease in the parotid gland with a poor prognosis in most cases. The disease most often develops in the setting of neurofibromatosis type 1 (NF1) but can also occur sporadically. Herein, we report a rare case of MPNST in the parotid gland, in a patient with no previous history of NF1. Initial investigations of the patient, which consisted of laboratory investigations, ultrasound imaging of the swelling, fine-needle aspiration (FNA), computed tomography (CT) scan, and magnetic resonance imaging (MRI) of the neck and swelling, were compatible with a benign pleomorphic adenoma of the parotid gland. However, intraoperatively, the dissection was challenging as the tumor was adherent to the neighboring tissue. A diagnosis of MPNST arising from a diffuse neurofibroma was established based on clinicopathologic features of the disease. The patient, who exhibited clinical features compatible with (NF1), proceeded for radiotherapy following surgery to continue his treatment.

## 1. Introduction

Malignant peripheral nerve sheet tumors, MPNSTs, are rare spindle cell sarcomas, accounting for 10% of all soft tissue sarcomas [1]. Most MPNSTs occur in the extremities and the trunk, while only 10% develop in the head and neck region [2]. We present a case of low-grade MPNST, arising in a diffuse neurofibroma of the parotid in a patient with no past history of neurofibromatosis type I (NF1). The clinical, histopathologic, and therapeutic aspects of this lesion are detailed in this report.

## 2. Case Presentation

A 45-year-old male from the Netherlands presented with a painless right parotid swelling that was progressively increasing in size for the past 8 months. Though occasionally he suffered from jaw lock, other symptoms associated with neurologic deficit such as drooling, facial weakness, paresthesia, or auditory defects were absent. Apart from being a social alcohol consumer, there was no history of smoking, prior radiation, or significant family medical history, especially in regard to his present illness.

**Figure 1:**
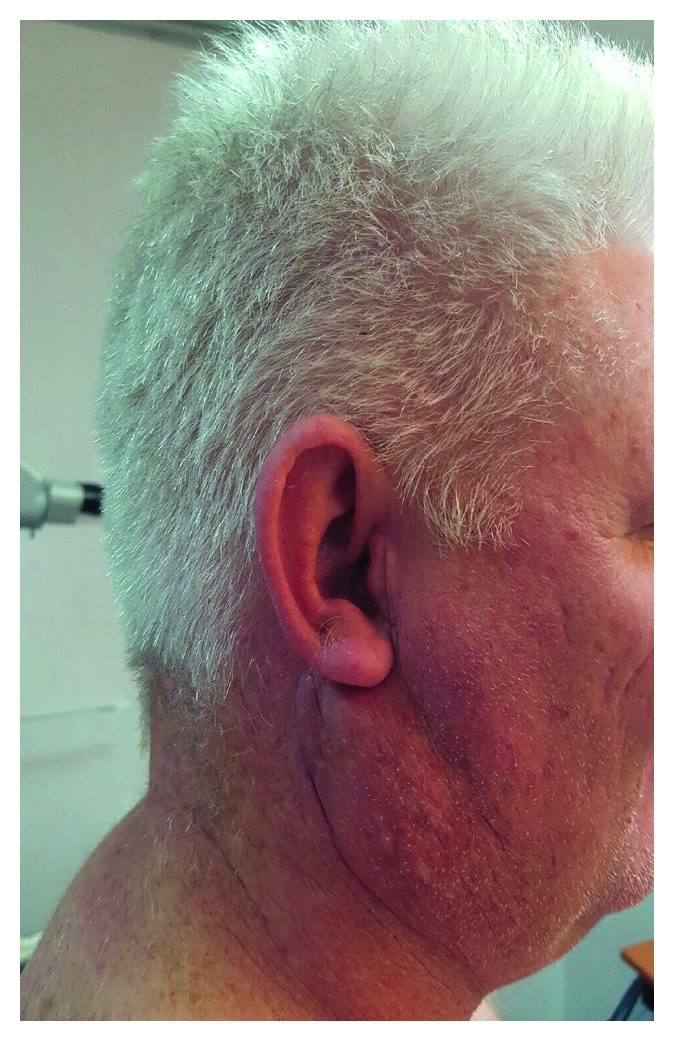
Preoperative clinical photograph showing a right parotid swelling.

Physical examination revealed a tender right parotid swelling below the ear lobule, which extended inferiorly to the angle of the mandible (Figure 1). The skin overlying the swelling was slightly erythematous, thickened, and nodular. The swelling was firm, diffused, and fixed to the underlying muscles, and there was no associated lymphadenopathy. Otoscopic examination of both ears was within normal limits.

**Figure 2:**
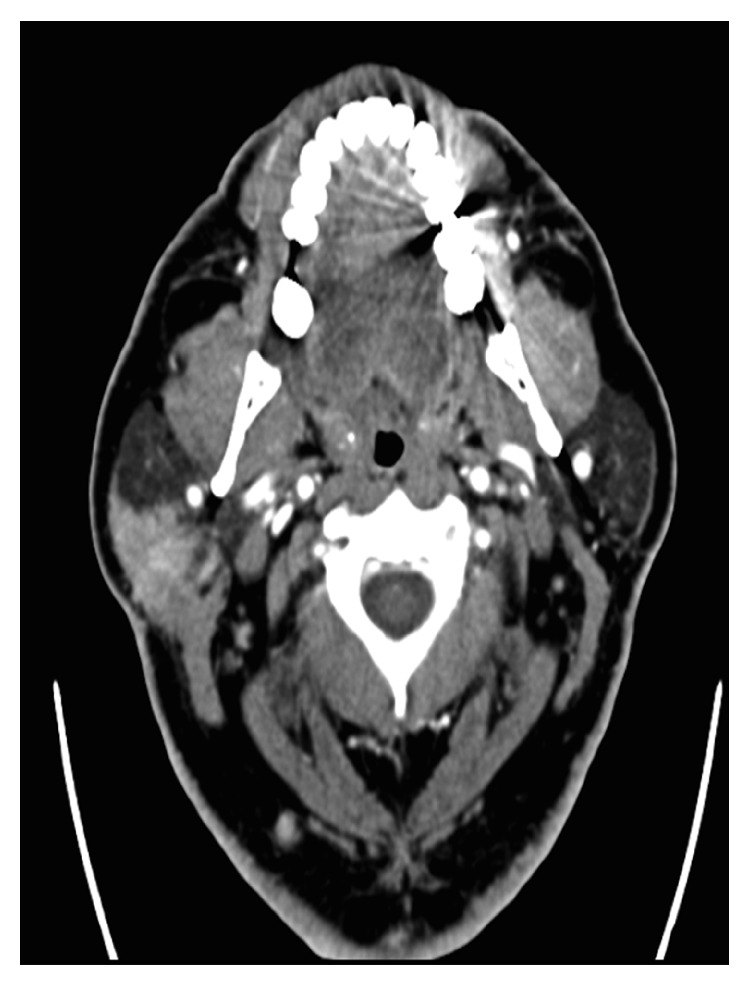
Axial CT with contrast shows a mass in the superficial lobe of the right parotid gland with post-contrast enhancement.

The patient initially had a neck ultrasound and then a magnetic resonance imaging (MRI) to characterize the nature of the lesion. The neck CT scan revealed a well-defined altered signal enhancing mass measuring 3.5 × 2.2 × 2.0 cm at the posterior aspect of the superficial part of the right parotid gland (Figure 2). The radiologist's impression was an altered signal enhancing mass lesion, likely to be a benign pleomorphic adenoma. The patient then underwent MRI of the parotid glands, which showed a well-defined focal lesion of altered signal intensity at the posterior aspect of the superficial part of the right parotid gland, measuring 3.5 × 2.2 × 2.0 cm along its maximum transverse, craniocaudal, and anteroposterior diameters, respectively. The impression was again benign pleomorphic adenoma of the right parotid gland. However, the radiologist could not exclude other diagnostic possibilities and recommend fine-needle aspiration cytology.

Fine-needle aspiration cytology of the mass was nonconclusive as the smears only showed polymorphous population of lymphoid cells in keeping with intraparotid lymph node. In view of these clinical findings, a superficial parotidectomy with facial nerve monitoring and preservation was planned.

**Figure 3:**
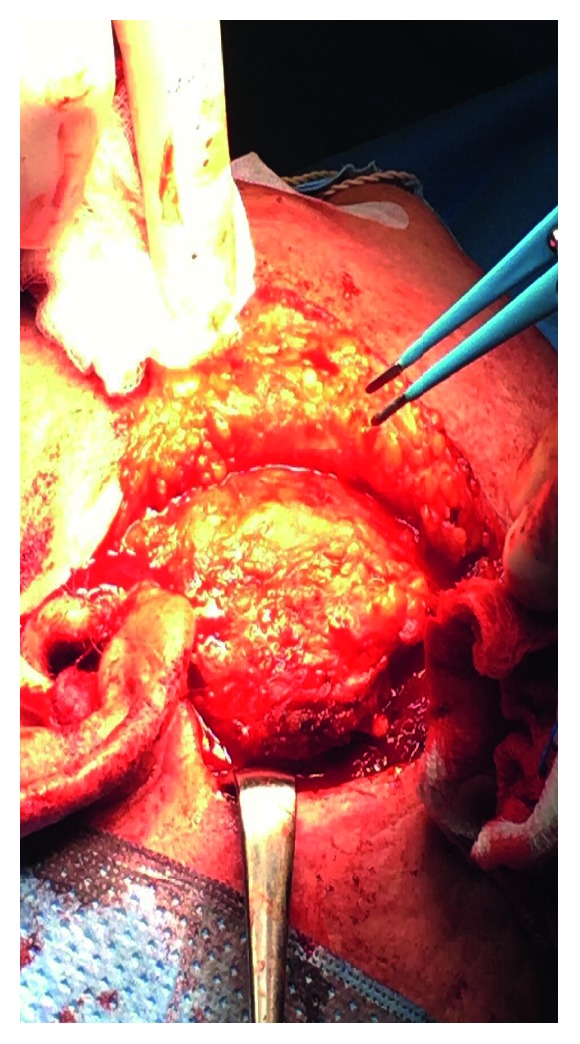
Clinical photograph showing intraoperative view of the parotid swelling.

A modified Blair incision was done, with elevation of a skin flap and control of hemostasis during the surgery. The dissection was rather difficult! The superficial parotidectomy was done in piecemeal as the mass was unexpectedly adherent to the skin and underlying fascia (Figure 3). The facial nerve was intact and was examined branch by branch. No frozen section was done during the operation.

**Figure 4:**
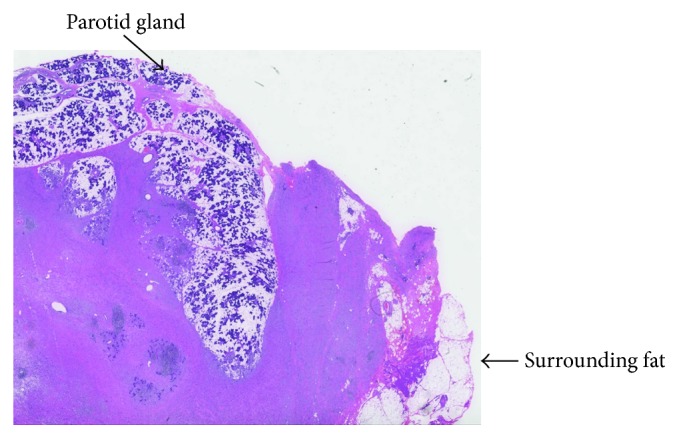
Histologically, diffuse-type neurofibroma arising in the parotid region with diffuse infiltration between salivary gland lobules and surrounding adipose tissue (arrows).

**Figure 5:**
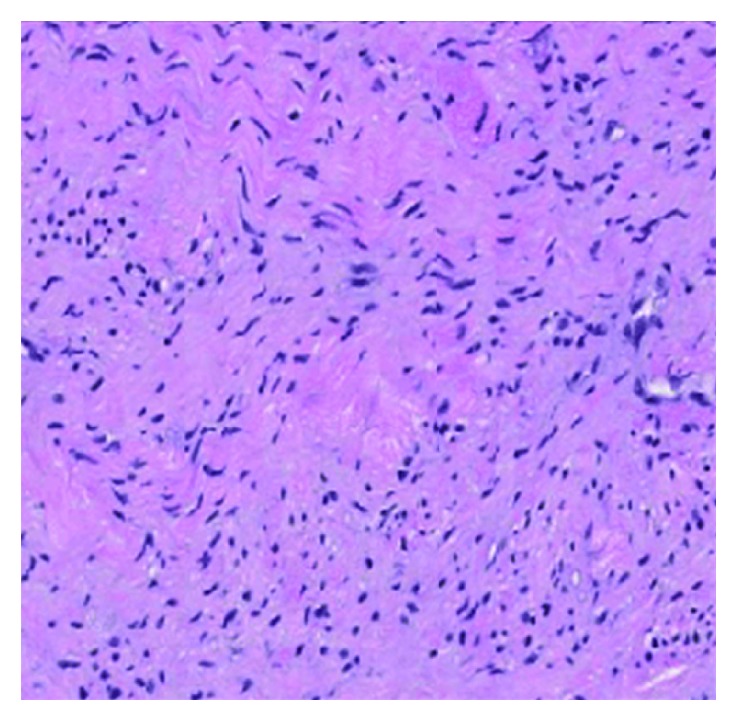
Diffuse-type neurofibroma comprises tumor cells with round to fusiform nuclei in a fine fibrillary collagenous matrix.

**Figure 6:**
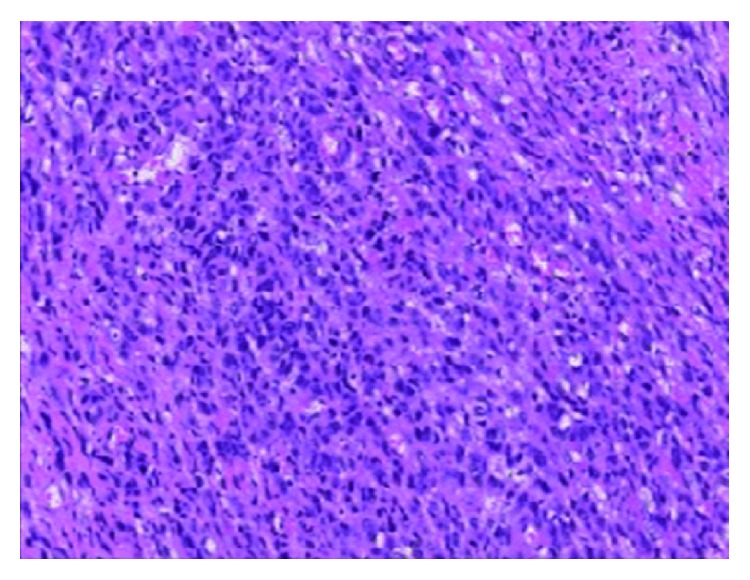
Areas of increased cellularity with epithelioid cells showing nuclear atypia and mitotic activity indicate transition to MPNST.

Histopathologic examination showed a widely infiltrative tumor involving the parotid parenchyma and extending into the surrounding adipose tissue and skeletal muscles (Figure 4). The tumor was predominantly composed of bland spindle cells with wavy nuclear contours, embedded within a fibrillary, pale pink matrix, which resembled a neurofibroma (Figure 5). Of concern, there were evidently distinct scattered more cellular foci of epithelioid tumor cells with nuclear atypia and increased mitotic activity (Figure 6). The tumor was diffusely and intensely positive for the S100 protein and neuron-specific enolase (NSE) immunohistochemical stains, with a low Ki-67 proliferation index. In addition, the CD34 immunohistochemical stain was focally positive in tumor cells. On the other hand, the tumor lacked immunoreactivity for AE1/AE3, p63, EMA, Melan-A, and HMB-45 immunohistochemical stains, thus excluding carcinomas, clear-cell sarcoma, melanoma, and other salivary gland tumors. Based on morphologic and immunophenotypic features of the tumor, the diagnosis rendered was low-grade malignant peripheral nerve sheath tumor (MPNST) arising in a diffuse neurofibroma.

**Figure 7:**
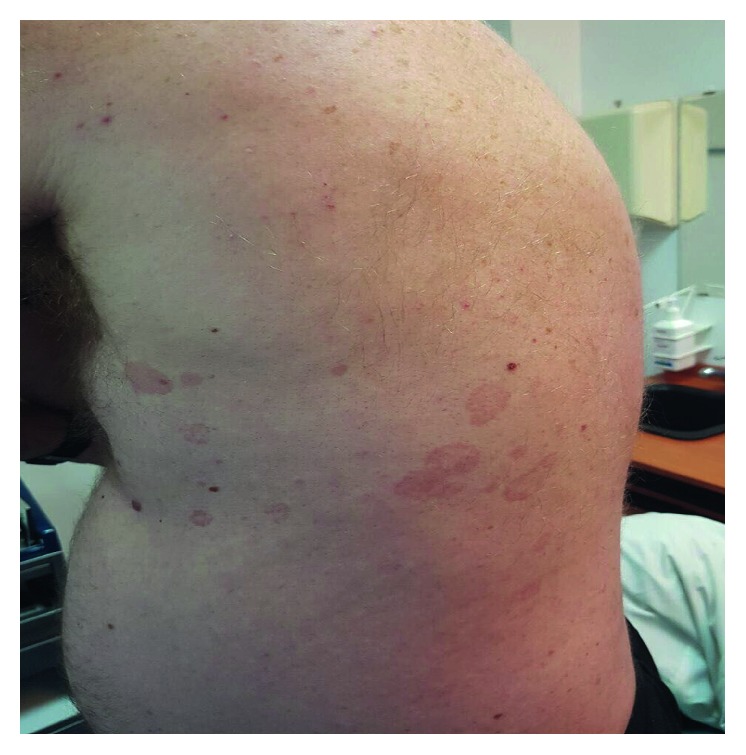
Clinical photograph showing multiple café au lait macules on the patient torso.

Given the rare presentation and the association of such tumors with NF1, the patient was evaluated clinically for features of the syndrome. Multiple café au lait macules were subsequently discovered on his trunk (Figure 7). The patient was referred to a cancer center for further management and follow-up.

Further clinical examination of the patient and MRI revealed no distant metastasis. The patient underwent several courses of radiotherapy and is currently eighteen months' disease free.

## 3. Discussion

Mesenchymal tumors constitute about 2% to 5% of all salivary gland tumors, most of which (>95%) involve the major salivary glands [3]. Thus, tumors of the peripheral nerve sheath origin presenting as intraparotid masses are even rarer and, when present, are likely to be benign. About one-half of MPNSTs are associated with neurofibromatosis type 1 (NF1) with tendency to arise from preexisting neurofibromas [4]. Sporadic cases of MPNST mostly occur in early middle age, between 30 and 50 years, but a younger age at presentation is usually seen in cases associated with NF1. Most case series show no obvious gender predilection for this disease. Of note, it is apparent that radiation therapy is a risk factor as patients with a history of irradiation are at an increased risk for developing this tumor [5].

MPNST most commonly presents as a mass and is often associated with pain; some patients even experience neurological symptoms, such as weakness or tingling. Sudden enlargement or worsening pain in a preexisting neurofibroma may indicate a malignant transformation to MPNST. These lesions are difficult to diagnose clinically given their rarity and nonspecific clinical presentation. MRI is the preferred choice of imaging modality, which guides diagnosis and assesses tumor extension [6]. Moreover, fine-needle aspiration cytology and histologic tissue examination are necessary for definitive diagnosis [6]; however, even some of these modalities have their limitations and challenges, as was seen in our case.

The histologic features of diffuse neurofibroma involving the parotid region are similar to those seen in diffuse neurofibromas of the skin or other sites. These include an infiltrative growth pattern characterized by extension between salivary gland lobules to the overlying or adjacent skeletal muscle, fascia, and adipose tissue. The tumor consists of bland spindle-shaped cells with ill-defined cell border and fusiform or wavy nuclei [4]. These cells are embedded in a fine fibrillary collagenous matrix, with occasional scattered Meissner corpuscles (eosinophilic lamellar structures) [4]. The histologic diagnosis of low-grade MPNST arising in diffuse neurofibroma is not straightforward. There seems to be a morphologic spectrum between neurofibroma, “atypical” neurofibroma, and low-grade MPNST [4]. No absolute cutoff criteria between these categories have been defined of as yet, making it very difficult to distinguish between them. Most of the low-grade MPNST cases described have 5 or fewer mitotic figures per 50 HPF [4, 7].

The majority of cases of MPNST have spindle cell morphology, with only 5% of cases showing epithelioid morphology [4]. In our case, the majority of the tumor, however, has characteristic histomorphologic features of diffuse-type neurofibroma with rare areas showing transition to frank MPNST, characterized by increased cellularity, nuclear atypia, and epithelioid cytomorphology. Although epithelioid MPNST is regarded as high grade by default, our case was designated as low grade because the epithelioid foci were scant and the bulk of the tumor was low grade in fact.

MPNSTs behave aggressively with frequent distant metastasis and local recurrence [6]. These tumors mostly spread through hematogenous route to the lung, bone, and liver [6]. Therefore, it is crucial that these patients receive prolonged clinical follow-up [6]. Overall, the behavior of MPNSTs depends on a number of factors including tumor location, size, stage, histologic grade, resection quality, necrosis, and association with NF1 [6, 8]. The standard treatment of all sarcomas is a complete surgical excision with clear margins. However, given the complex nature of the head and neck anatomy, complete excision is not always possible [1]. An alternative route would thus be excision combined with high-dose radiotherapy [1], as was done for our patient. It has been described that adjuvant radiotherapy is beneficial in tumors that are high grade, large in size, or with positive surgical margins [6]. There are no randomized studies assessing the role of adjuvant chemotherapy specifically for MPNST [8]. Therefore, the role of adjuvant chemotherapy in the treatment of MPNSTs is debatable and still under assessment [5, 6].

This was a challenging case due to the fact that all preoperative investigations pointed toward a benign entity, namely, pleomorphic adenoma. The rapid increase in size of the tumor, despite the preoperative investigations pointing to a benign disease process, was still worrisome for malignant transformation, especially with the appearance of the overlying skin and the lack of a reason for such a sudden and progressive increase in size (e.g., cystic formation/degeneration due to trauma). The suspicion for malignancy increased tremendously during surgery because of tumor adherence to the surrounding tissue rendering the superficial parotidectomy and tumor dissection very difficult.

## 4. Conclusion

MPNST is an extremely rare disease in the parotid gland. And when it is low grade, the diagnosis of malignancy can be very difficult and only possible with close examination and correlation of all available data, and histomorphologic, radiologic, and clinical features. In this manuscript, we presented a case of MPNST in the parotid gland, in which all preoperative investigations were suggesting a benign pleomorphic adenoma. But the histologic examination leads to a diagnosis of malignancy and discovering a syndromic association.

The patient underwent postoperative radiotherapy and is currently eighteen months' disease free! Clinicians should be aware of the association of MPNSTs and diffuse neurofibromas with NF1, a diagnosis, if confirmed, with great clinical and genetic implications for the patient and his/her family.
